# Genomics alterations of metastatic and primary tissues across 15 cancer types

**DOI:** 10.1038/s41598-017-13650-3

**Published:** 2017-10-16

**Authors:** Gang Liu, Xiaohui Zhan, Chuanpeng Dong, Lei Liu

**Affiliations:** Shanghai Public Health Clinical Center and Institutes of Biomedical Sciences, Fudan University, Shanghai, P. R. China

## Abstract

Metastasis is an important event for cancer evolution and prognosis. In this article, we analyzed the differences in genomic alterations between primary and metastatic tissues at hotspot regions in 15 cancer types and 10,456 samples. Differential somatic mutations at the amino acid, protein domain and gene levels, mutational exclusiveness, and copy number variations were identified in these cancers, while no significant nucleotide and gene fusion differences were detected. The homogeneity and heterogeneity of these differences in cancers were also detected. By characterizing the genomic alterations of these genes, important signaling pathways during metastasis were also identified. In summary, the metastatic cancer tissues retained most genomic features of the primary tumor at the biological level and acquired new signatures during cancer cell migration.

## Introduction

Tumor metastasis is among the most deadly consequences of cancer development, whereby cancer cells populate a new organ and flourish to ultimately cause dysfunction of the new tissue^1^. The lineages of the cancer cells in a tumor underlie the genomic heterogeneity of cancer. Some lineages expand their population, and others colonize distant tissues by migrating through the lymph or circulatory systems, as an indication of the evolutionary success of the individual lineage. Although less than 0.01% of cancer cells develop into metastatic tumors based on animal models^2,3^, the population of cancer patients with distant metastases is large.

Molecular alterations in various cancers have been investigated to elucidate the potential mechanism of cancer metastasis. In breast cancer, genes including LOX, FGFR, EREG, COX-2, and CXCR4 were shown to trigger metastasis initiation, progression and virulence. Some of these genes cooperate to remodel the vasculature and thus promote metastasis^4^. Chromosome 18 amplifications, chromosome 17 losses and ras mutations are increased during colorectal tumor development^5^.

Comparison of genomic alterations between different categories is the most frequently implemented method for studying potential mechanisms of metastasis. However, this method demands a large sample size and good-quality data to ensure the accuracy of the results. Although, the Cancer Genome Atlas (TCGA) has provided genomic data for cancer samples, the metastatic sample data are still lacking. AACR Project Genomics Evidence Neoplasia Information Exchange (GENIE)^6^ has collected the genomic data in hotspot sites of 18,966 cancer samples from both primary and metastatic tumors, and these data were collected from eight centers worldwide. Recently, GENIE has publicly released these data, making it possible to compare genomic alteration differences between primary and metastatic tissues.

Using publicly released data, we analyzed 10,456 samples from 15 cancer types. Significantly different genomic mutations, copy number variations, and gene fusions in hotspot regions were compared between the primary and metastatic tumor tissues in these cancer types. Genomic heterogeneity and homogeneity were analyzed among cancers. By integrating the genomic alterations, we identified altered signaling pathways associated with metastasis.

## Results

### Clinical characteristic overview of samples

In total, 10,456 samples were included in this study. The hotspot regional mutations and copy number variations of these samples were available from GENIE. Among these samples, gene fusion data from Memorial Sloan Kettering Cancer Center (MSK) were used for further analysis due to the panel size and data availability. Finally, 4472 samples were enrolled in this step.

**Figure 1:**
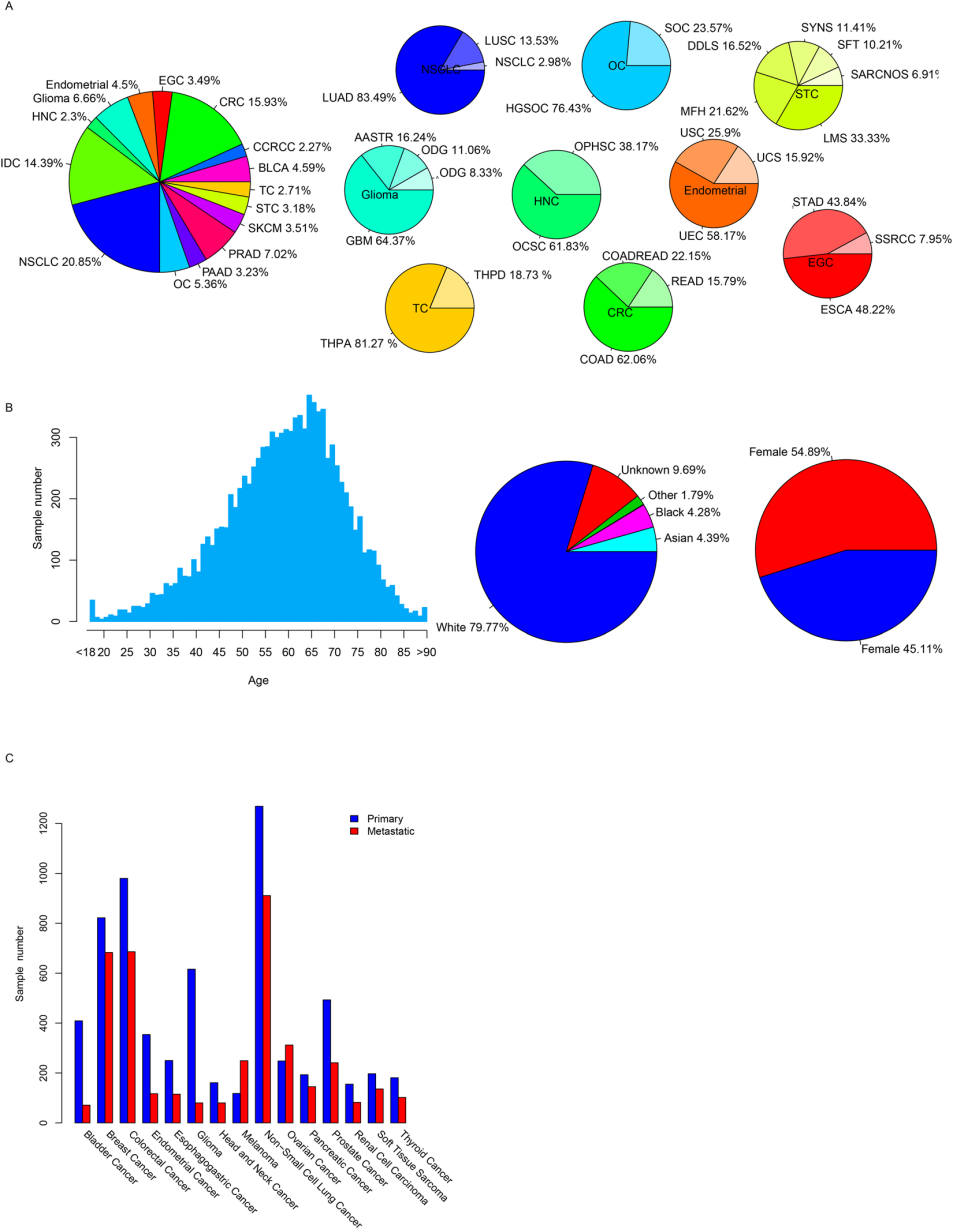
Sample distributions in categories. The distribution of cancer types (**A**), age, gender, race (**B**), and primary/metastatic tissues (**C**).

According to the information provided by GENIE, we divided samples into 15 broader cancer types (Fig. 1A). The cancer categories containing the most samples were non-small cell lung cancer (NSCLC, 20.85%), colorectal cancer (CRC, 15.93%), breast invasive ductal carcinoma (IDC, 14.39%), prostate cancer (PRAD, 7.02%), and Glioma (GBM, 6.66%). Among these samples, metastatic cancer accounted for at least 14.79% of samples in each cancer type (Fig. 1B), and 67.85% of melanoma samples were metastatic samples. For gender information, 54.89% were female and 45.11% were male (Fig. 1C). This bias was introduced by the gynecological cancer samples, including breast cancer and ovarian cancer. Most of the samples included in this study were obtained from Caucasians (79.77%, Fig. 1D), which would be explained by the center locations of GENIE. The ages of the patients ranged from 40–80 (9303/10456, 89.03%, Fig. 1E), and the median age was 62. Detailed information of sample statistics is provided in Table S1.

### Mutational landscape of hotspot genes in primary and metastatic cancers

**Figure 2:**
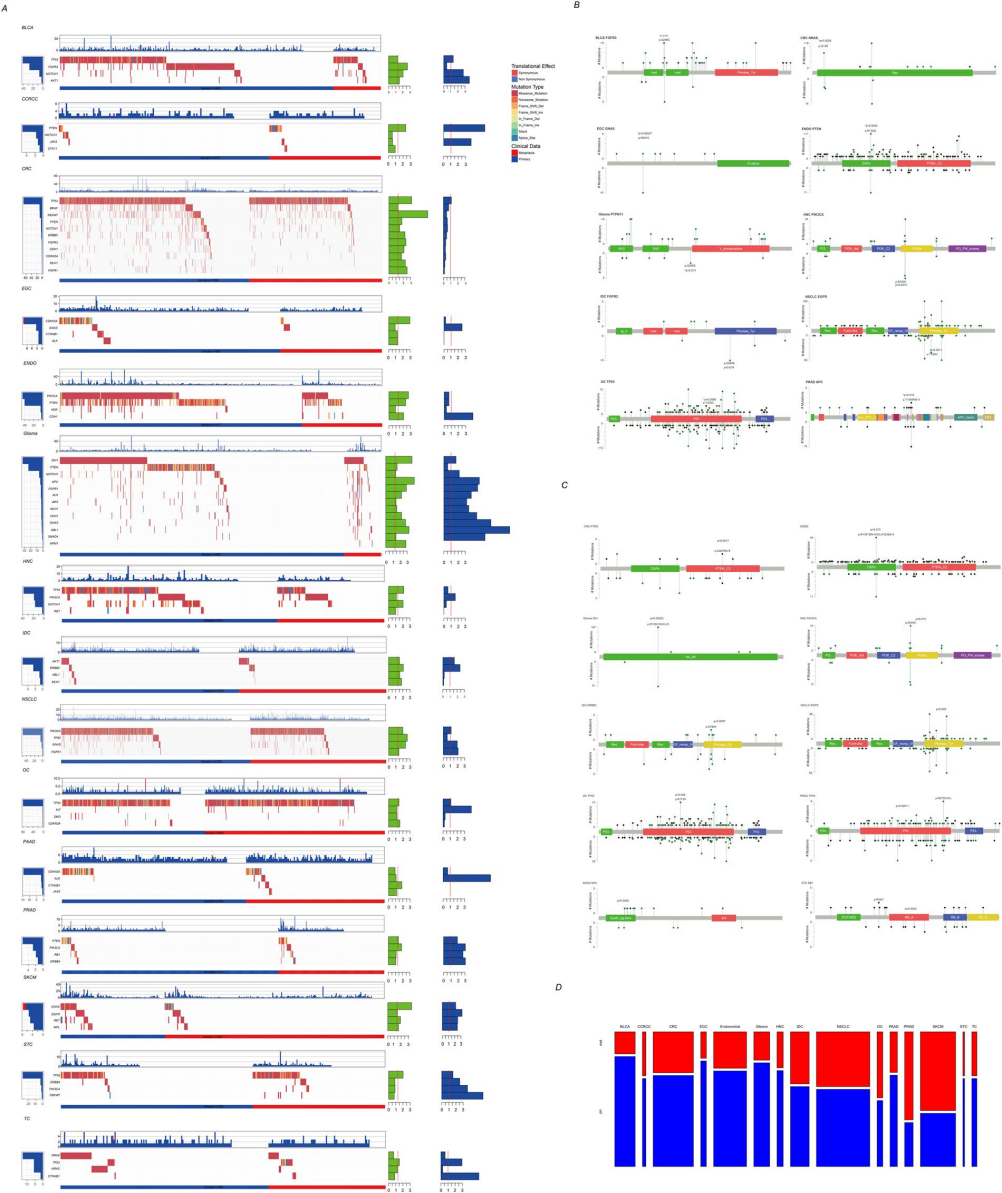
Mutational difference of primary/metastatic tissues. The differentially mutated genes in cancer types (**A**). The left panel indicates the mutational rate in metastatic and primary cancerous tissues; the top panel refers to the mutational frequencies of each sample in regions detected; the green barplot in the right panel represents the log2 transformed p values of mutational difference between metastatic and primary tissues; the blue barplot in the right panel indicates the mutational odds ratio of metastatic/primary mutation rates; the middle panel refers to the mutation type of each sample in each gene. The mutational difference between primary and metastatic tissue on each amino acid was shown in needle plot (**B**). The height represents the mutational counts in different regions and domains, the needles upward indicates the primary and needles on the other side refers to metastatic tissues. The mutational difference between primary/metastatic tissue was also shown (**C**). Mutational exclusive pairs in primary/metastatic tissues in each cancer type (**D**). The width of each bar indicates the pair number and the height indicates the proportion.

We first analyzed the genomic mutations of hotspot regions at the gene level across 15 cancers in both primary and metastatic tissues and compared the mutational differences between primary and metastatic sites (Fig. 2A). Among these genes, the TP53 mutation rate of metastatic cancer was significantly higher in six different types of cancers (BLCA, CRC, NSCLC, OC, STC, and TC) but lower in HNC, compared to the primary tissue (Fig. 2A and Table 1). Mutation of PTEN was significantly different in five cancer types, among which the mutation rate of PTEN in ccRCC and PRAD was higher in metastatic cancer but lower in CRC, Glioma, and ENDO. The mutation of other genes including NOCTH1, PI3KCA, GNAS, MLH1, FGFR1, CDKN2A, CDH1, ABL1, ERBB2, FGFR3, and AKT1 was also significantly different between primary and metastatic tissues across cancers. Mutation of thirteen genes in Glioma was significantly different between metastatic and primary sites, and the mutation rate of these genes in metastatic tissue was higher compared to the primary site, except for PTEN. Significant genomic alterations of eleven genes were also observed in CRC. However, in contrast to Glioma, the mutation rate for most genes was significantly lower in metastatic tissues, except for TP53 (44.72% vs 26.26% for primary and metastatic).

**Table 1:** Genes mutation rate in pirmary and metastatic tissues across cancers.

Gene	Mutation rate lower in metastatic tissue	Mutation rate higher in metastatic tissue	sum
TP53	HNC	BLCA,CRC,NSCLC,OC,STC,TC	7
PTEN	CRC,ENDO,GBM	CCRCC,PRAD	5
NOTCH1	CRC,HNC	BLCA,GBM	4
CDKN2A	CRC,EGC,OC	None	3
MLH1	CRC,IDC	GBM	3
PIK3CA	ENDO,NSCLC	PRAD	3
CDH1	CRC	ENDO,GBM	3
FGFR1	CRC	NSCLC,GBM	3
GNAS	SKCM	NSCLC,GBM	3
FGFR3	BLCA,CRC	None	2
ERBB2	CRC	IDC	2
ABL1	IDC	GBM	2
AKT1	None	BLCA,IDC	2

In addition, we noticed that the mutational exclusiveness existed across cancer types in both primary and metastatic cancers (Table S2). Although exclusive pairs in the primary tissues were much higher than in the metastatic tissues (Fig. 2B) and a half exclusive mutational pairs in metastatic tissues were also observed in the primary pairs (49.70%, 84/169), metastatic-specific mutational exclusive pairs were also identified (Table S3). For example, ALK-BRAF and ERBB4-BRAF exclusiveness was detected in metastatic SKCM (p = 0), but not in primary tissues. Similarly, EGFR-JAK3 and CTNNB1-TP53 exclusiveness was observed only in NSCLC metastatic tissues (p = 2e-4, 3.1e-4, respectively). Homogeneity of mutational exclusiveness was also observed in cancers. For example, PIK3CA-TP53 mutational exclusiveness was detected in both metastatic and primary tumor tissue in seven cancer types, among which, BLCA, CRC, ENDO, Glioma, HNC, IDC were found to be exclusive in both primary and metastatic tissues.

The mutational difference between primary and metastatic tissues on each gene was then investigated to identify the potential metastatic driver sites. The nucleotide mutation contents of the regions were then compared between metastatic and primary tissues, but no significant mutation content differences were detected (Fig. S1). Amino acid alterations were also compared, and 20 amino acid-cancer pairs were detected in 10 out of 15 cancers (Fig. 2C, Table S4). Amino acids on TP53 were also detected to have more significantly different mutation sites between primary and metastatic sites in four cancer types (CRC, Glioma, NSCLC, and PRAD) on eight sites. In addition, the IDH1.R132H, GNAS.R201C, and EGFR.T790M mutations in Glioma, EGC, and NSCLC were the most significantly different point mutations (p = 0.00021, 0.00027 and 0.0011, respectively). In addition, PIK3CA.E542K, PTPN11.G268S, AKT1E17K and APC.T1556Nfs*3 were also observed in multiple cancer types.

The protein mutational rate differences in protein domains were as also compared between metastatic and primary tissues (Fig. 2D, Table S5). Among the 1508 gene-domain pairs according to PFAM, 52 was identified as significantly different. We detected significantly altered protein-domain pairs in ten cancer types, in which the p53 domain of TP53 in PRAD (p = 0.00011), Iso_dh domain of IDH1 (p = 0.00022) and Gly_rich domain of ALK (p = 0.00074) in Glioma were the most significantly altered. In addition, the Pkinase domain of AKT1 in BLCA, DSPc domain of PTEN in CCRCC, Pkinase_Tyr domain of ERBB4 in EGC, DSPc domain of PTEN in ENDO, PI3Ka domain of PIK3CA in HNC, Pkinase_Tyr domain of ERBB2 in IDC, Pkinase_Tyr domain of EGFR in NSCLC, and P53 domain of TP53 in OC were also detected to be significantly associated with metastasis. Most of these differential mutations were enriched in metastatic tissues, except for PTEN_C2 in CRC, DSPc in ENDO, and EpoR_lig-bind in SKCM.

Collectively, despite the vast majority of the mutational variations in metastatic tissue being similar to the original tissue, there were still significantly differently mutated genes, amino acid sites, protein domains and mutational exclusiveness that existed across cancer types.

### Copy number variation in tissue types

**Figure 3:**
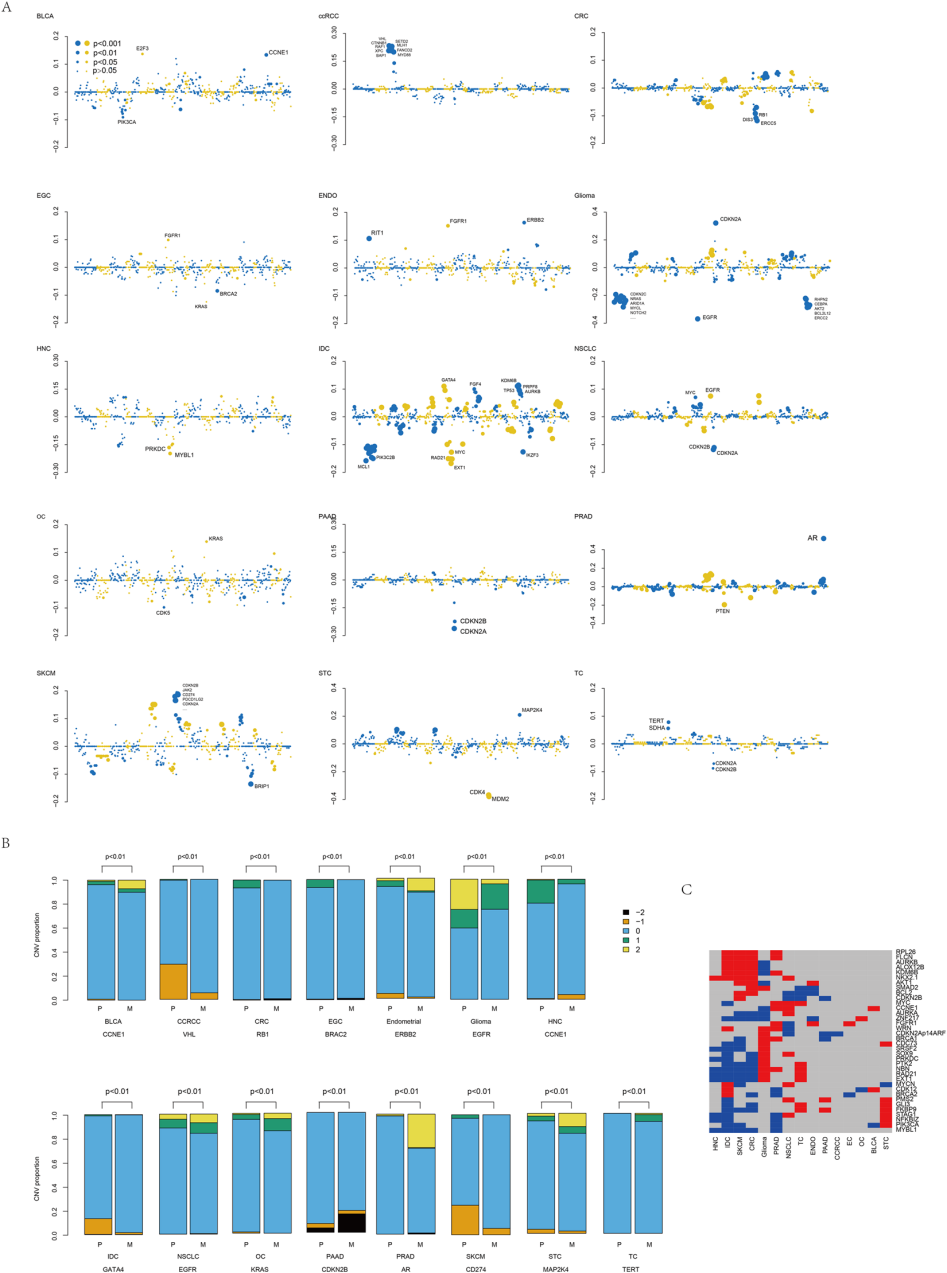
Copy number difference of primary/metastatic tissue. Average copy number difference between metastatic and primary tissues (**A**). The y-axis indicates average copy number of metastatic tissue - average copy number of primary tissue, and x-axis refers to chromosomal locations (chr1-chrY). The detailed copy number distribution of most significantly different genes in each cancer type was shown (**B**). Most significantly altered genes in metastatic/primary tissues across cancers. The red indicates copy number increased in metastatic tissues and blue refers to decreased.

The copy number variation differences between primary and metastatic tissues across cancers were also investigated in 610 frequently amplified/deleted regions. Homogeneity and heterogeneity in the copy number alteration levels of cancer types were observed between primary and metastatic tissues (Fig. 3A–C). A significantly altered copy number variation between primary and metastatic cancers was detected in all 15 cancers, especially Glioma, IDC, SKCM, PRAD, CRC and NSCLC. Cell cycle genes, including CDKN2A and CDKN2B, were detected to be frequently different between primary and metastatic tissues (Table S6). 17p13.1 was a significantly enriched altered region in CRC, Glioma, IDC, PRAD, and SKCM (Table S6). We noticed that the copy numbers of E2F3 and CCNE1 were increased, while PIK3CA was decreased in BLCA. Genes on cytoband 3p (VHL, CTNNB1, SETD2, BAP1, etc.) were significantly enhanced in ccRCC. Decreased RB1, ERCC5, and DSP3 in CRC, amplification of FGGR1 and deletion of BRCA in EGC, an increased copy number of RIT, FGFR1 and ERBB2, decreased MYBL1 and PRKDC in HNC, alteration of KRAS and CDK5 in OC, alteration of CDKN2A/B in PAAD, amplification of AR in PRAD, copy number decreases in MDM2 and CDK4 in STC, and increased TERT and SDHA in TC were detected in metastatic cancer compared to the original tissues. Among these genes with significantly altered copy numbers between primary and metastatic cancers, it was noted that the copy number of CDKN2A/B was decreased in metastatic cancer in TC, PAAD and NSCLC but increased in SKCM and Glioma, suggesting that the role of CDKN2A/B is different in the metastasis in cancers.

### Gene fusion landscape comparison of primary and metastatic tissues

Gene fusion has been shown to be an important cause of carcinogenesis, but its function in cancer development and metastasis is still unclear. Thus, we analyzed gene fusions in these cancer types and investigated 341 hotspot gene fusion differences in 4472 samples (detailed sample information detected gene fusion is provided in Table S7) provided by Memorial Sloan Kettering Cancer Center (MSK) using GENIE. First, we investigated the gene fusion pairs across cancer types. In contrast to somatic mutations, the gene fusion rate across cancers is relatively rare. The highest fusion rate was the TMPRSS2-ERG fusion in PRAD, with 21.63% in primary cancer and 22.34% in metastatic cancer (Fig. S2, Table S8). The gene fusion rates of primary and metastatic tissues across cancer types were compared, and none was significantly different. Single fusion genes were also compared between metastatic and primary tissues (Not shown). Coincident with the fusion pairs, none of these genes was significantly different between primary and metastatic tissues (Fig. S3). These results indicate that gene fusion may have contributed carcinogenesis, but its function in promoting cancer migration and metastasis may be limited.

### Genomic alterations of pathways during metastasis

**Figure 4:**
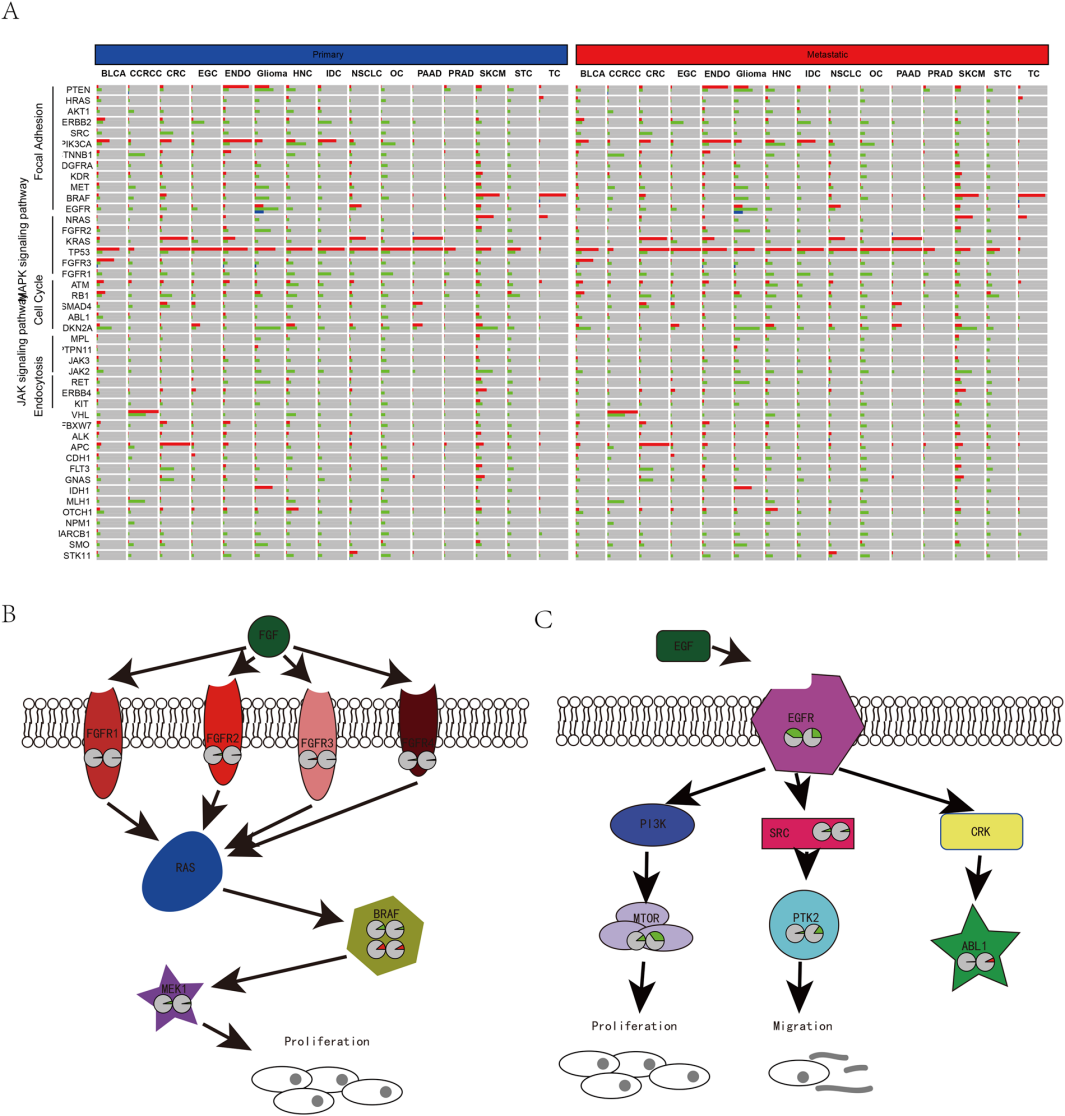
Altered genes and pathways in cancers. The integrated mutation, copy number variation and gene fusion rate in primary and metastatic tissues (**A**). Among KEGG pathways, we noticed that MAPK signaling pathway was significantly different genes involved in CRC (**B**) and ERBB signaling pathway in glioma (**C**). The red pie plots indicate the mutation rates and greens indicate copy number alteration rates. All genes with pie plots were statistically different between primary and metastatic tissues.

By combining genes involved in important cancer metastasis-related pathways, we found that these genes participate in the cell focal adhesion, cell cycle, MAPK, and JAK signaling pathways, especially focal adhesion, according to KEGG. Most genes in these pathways were significantly altered in different types of cancers (Fig. 4A). Specifically, we noticed that the alteration ratio of the MAPK signaling pathway was significantly different between metastatic and primary cancers in CRC (Fig. 4B). The genomic alteration rate of four fibroblast growth factor receptors (FGFR1, FGFR2, FGFR3, and FGFR4) was significantly lower in metastatic tissues. Among these FGFRs, the mutation rates of FGFR1 and FGFR3 were significantly lower in primary tissue compared to metastatic tissues. The copy number aberrancies of FGFR2 and FGFR4 were consistent with these results. In addition, the alteration of genes downstream including BRAF and MEK1 also supports this observation, and both the mutation and copy number aberrancies of BRAF were significantly lower in metastatic tissues. Genomic alteration differences for genes in the ERBB signaling pathway were also detected, and the results were more complex (Fig. 4C). The copy number alterations of upstream genes including EGFR and SRC were lower in metastatic tissue in contrast to the primary tissues, while genomic alterations of downstream genes, including copy number variations of MTOR and PTK2 and mutation of ABL1, were higher in metastatic tissues. This may be explained by the complex role of EGFR.

## Materials and Methods

### Sample enrollment and raw data

GENIE v1.0 provided the mutation, copy number variation and gene fusion information of 18,966 tumor samples from 478 onco-types. Most onco-types were classified into 15 categories (detailed information regarding sample, onco-type, and primary and metastatic tissue number are shown in Fig. 1A and Table S1) according to Oncotree (http://oncotree.mskcc.org/oncotree/), as non-small cell lung cancer (NSCLC), breast cancer (IDC), colorectal cancer (CRC), prostate cancer (PRAD), Bladder cancer (BLCA), Glioma, ovarian cancer (OC), pancreatic cancer (PAAD), melanoma (SKCM), ENDO Cancer (Endometrial), renal clear cell carcinoma (CCRCC), thyroid cancer (TC), esophagogastric cancer (EGC), soft tissue sarcoma (STC), and head and neck cancer (HNC). Onco-types not included in these 15 categories were excluded in this study.

Raw data were downloaded from Synapse (syn7222066, https://www.synapse.org/) and provided by the GENIE project using R commands. The preprocessing protocols for these data are described in the GENIE-provided data guide.

### Mutation analysis

Since the gene panel sizes in the assays and centers were not identical, we only selected the common genes for further analysis in all assays and centers for each cancer type (genes for each cancer type are listed in Table 9), and only exon regions were retained. Nonsense, missense, splicing site, stop-gain, out-of-frame small, and in-frame indel mutations were retained as somatic mutations, while the other mutational information was excluded in this study. Differential mutation of each gene/site was’ evaluated with mutational information in primary and metastatic tumors using Fisher’s exact test (p < 0.05 as statistically significant). Protein domains were downloaded from the pfam website^7^ (http://pfam.xfam.org/), and differentially mutated protein domains were also assessed with the same method (p < 0.05). The mutational rate of nucleotides was assessed as

$$\mathrm{Mutation}\_\mathrm{per}\_\mathrm{MB}=\mathrm{mutation}\_\mathrm{total}/\mathrm{coverage}\_\mathrm{space}\ast {\rm{1000000}}$$

where mutation_total refers to the total mutation number in a specified region for each sample, and coverage space indicates the total exon region for each cancer type. The mutational landscape and transition-transversion plot were visualized with R package “GenVisR”^8^. Differentially mutated sites of amino acid alterations and differentially mutated protein domains were plotted using MutationMapper (http://www.cbioportal.org/mutation_mapper.jsp) in cbioportal^9^.

WESME was used to assess the mutational exclusiveness for each cancer type. After 1000 permutations, exclusive p-values were calculated for each gene pair and each cancer type, and p < 0.05 was considered statistically significant.

### Copy number variation statistics

Copy number alteration data were available at AACR Project GENIE, https://synapse.org/genie, which collected more than 10,456 samples from eight different centers. In the present study, we selected the 15 most common cancer types for consideration. The Cochran-Armitage Trend test was used to calculate the difference in the CNA between the primary and metastatic tumors (using the “DescTools” package) and p < 0.05 was regarded as statistically significant. Meanwhile, we calculated the changes in the average copy number between primary and metastasis samples. R was used to plot the change in the number of copies of the gene in different chromosomes based on cancer type. The ordinate represents the difference value between the mean copy number for the metastasis samples and that of the primary tumor samples, and the size of the point indicates the statistical significance. Genes showing the strongest differences are displayed in the plot.

### Gene fusion processing

Raw data from the GENIE gene fusion were downloaded from Synapse, since only two centers, VICC (Vanderbilt-Ingram Cancer Center, USA) and MSK, have provided gene fusion information and gene panel sizes. The sample number from VICC was limited, so we only used MSK-provided data for further analyses. Since MSK contained two assays, consisting of 341 and 410 genes, we used the intersected genes in both assays for analyses (341 genes). Gene fusion rates for single-gene fusions and gene pairs were calculated. For visualization, R package “Rcircos”^10^ was used to plot circus plots using the hg19 genome build. A gene fusion matrix for each sample was constructed and plotted with R package “pheatmap”.

## Discussion

Clinically, metastasis is a pathological event that makes surgery difficult and often affects the survival of cancer patients. Biologically, metastasis is an evolutionary event in cancer, where cells migrate to new loci for colonization. Genomic alterations, including somatic mutations, copy number variations, and gene fusions, promote this event via different pathways involved in various cell processes. Thus, investigating the heterogeneity and homogeneity of genomic alterations during metastasis is important to provide a basis for cancer metastasis.

The solid tumor consists of cancer cells that originate from specific lineages and new genomic alterations occur with each passage. Some of the cells acquire new genomic features, separate from the original tissue, enter the lymph or blood vasculature, localize to the new tissue and colonize it to form another tumor. During this process, the metastatic tumor tissue originates from the primary site and retains most of the original genomic signatures. Since the metastatic site is originated from a single cell (lineage) of the primary tissue, the genomic features of the metastatic cell are less complex than the original site, and a part of the acquired genomic alteration also exists in the primary tumor tissue. However, the proportion of these cells in the primary tissue may be limited, causing the genomic alteration to be barely detected. This explains why some genomic alterations were enriched in the metastatic tissues. It has been widely reported that some signaling pathways contribute to carcinogenesis but suppress cancer migration. For example, XXX, and this explains how the mutations or copy number alteration rates of some specific genes in the metastatic tissues was relatively lower than the primary tissues.

Overall, differential mutation of somatic genes was detected at the gene, amino acid, and protein domain levels. Among these genes, TP53 and several other genes were recurrently, significantly, and differentially mutated at each level. Copy number variation was also detected among cancers, and recurrently, significantly, and differentially copied genes were also detected. However, differential gene fusion was not detected at the fusion pair or single-gene level, although the gene fusion rate in some cancers is high. The result indicates that mutation and copy number were involved in cancer metastasis while gene fusion was probably not involved. Reports showing that gene fusion promotes or suppresses migration or metastasis are limited, but it is not the case for copy number variation or mutation, which is consistent with our result.

Genomic alteration differences of primary and metastatic tissues may result from multiple reasons. For instance, alterations of some specific genes induce high metastatic ability, and thus these alterations may significantly higher in metastatic tissue. On the other hand, genomic alteration of some genes may result in short survival, and the proportion of these samples would be limited, causing these alterations barely detected. Among genomic mutations, TP53 is a well-known gene for suppressing both carcinogenesis and metastasis^11^ by influencing cell mortality^12^, the EMT (Epithelial mesenchymal transition)^13^, and interactions with the ECM^14^. Thus, the mutation and amino acid alteration rates of TP53 are enhanced in most metastatic tumors, compared to the primary tissues. We noticed that the TP53 mutation rate in HNC is reduced, and the mutation rate of the P53 domain is also significantly lower in the metastatic tissues. Although previous results have indicated that TP53 mutations are associated with a poor prognosis in HNC^15,16^, which is inconsistent with our results, there are no reports of investigations of the TP53 mutation rate in primary and metastatic tissues. We also noticed that the IDH1.R132H mutation rate was evaluated in metastatic tissues and compared to primary tissues (47.5% vs 27.6%). Interestingly, patients harboring the IDH1.R132H mutation had a relatively lower invasion rate, and the mutation was associated with good survival^17–19^. The survival time of wild-type IDH1 patients is approximately one year, according to a recent report^18^. Thus, we propose that IDH1.R132H was probably detected more in the metastatic tissue because none the GBM patients died early due to the primary tumor. EGFR.T790M has been reported as the most important feature of recurrent NSCLC^20–22^, and it is associated with drug resistance, while its role in metastasis has yet to be reported. According to our analysis, the mutation ratio for metastatic tissue is significantly higher in metastatic tissue (4.94% vs 2.21%), suggesting that T790M is also associated with metastasis.

Carcinogenesis can initiate via distinct pathways. Thus, mutational exclusiveness was detected. During cancer metastasis, the wild-type pathways may be needed. Thus, some exclusive pairs disappeared, which explains why mutationally exclusive gene-pairs were reduced in the metastatic tissues, compared to the primary tissues. On the other hand, we hypothesize that metastasis can also occur via distinct pathways, and thus, new mutationally exclusive pairs were detected in metastatic cancer. Data that support this notion show that the most recurrent genes of the metastasis-specific genes identified were TP53, BRAF, and KRAS (23/14/7 pairs, respectively), and these genes are well-known metastasis-promoting genes^11,23–25^.

At the pathway level, we detected that the alteration rate (mutation and copy number variation) of the genes involved in MAPK signaling pathways, including FGFR1, FGFR2, FGFR3, FGFR4, BRAF, and MEK, was significantly lower in metastatic colorectal cancer tissue compared to the primary site. Among these genes, the BRAF mutation was reported to decrease liver-limited metastasis^24^. Fibroblast growth factor receptors were reported to have important roles in the tumor–stroma interaction, and FGFR4 was demonstrated to correlate significantly with tumor development and lymph node metastasis of colorectal cancer^26^. Alteration of MEK may promote metastatic progression of colorectal cancer via downstream ERK and AKT pathways^27^.

There are limitations in this study. We filtered the commonly existing genes among centers, and the protocols and pipelines used in these centers are proven to be robust. However, the most important limitation is that these results are based on different batches and different pipelines, especially for the mutation data. This may have introduced an important bias when analyzing the primary-metastatic tissue difference. Another limitation of this study is that this is a retrospective study. Sample enrollment was not strictly controlled, and important clinical information, including TNM staging, survival information, and therapy method, was not included.

## Electronic supplementary material

Supplementary figures

supplementary tables
