# Supplementary material for: Genomics alterations of metastatic and primary tissues across 15 cancer types
Source: Sci Rep. 2017 Oct 16;7:13262. doi: 10.1038/s41598-017-13650-3 (PMC5643378; doi:10.1038/s41598-017-13650-3)

Genomics alterations of metastatic and primary tissues across 15 cancer types

Gang Liu[1,*], Xiaohui Zhan[1,*], Chuanpeng Dong[1,*], Lei Liu[1,$]
[1]Shanghai Public Health Clinical Center and Institutes of Biomedical Sciences, Fudan University, Shanghai, P.R.China.
[*]These authors contributed equally to this work
[$]Corresponding author: liulei_sibs@163.com

Fig. S1. Mutational nucleotide content in cancers.

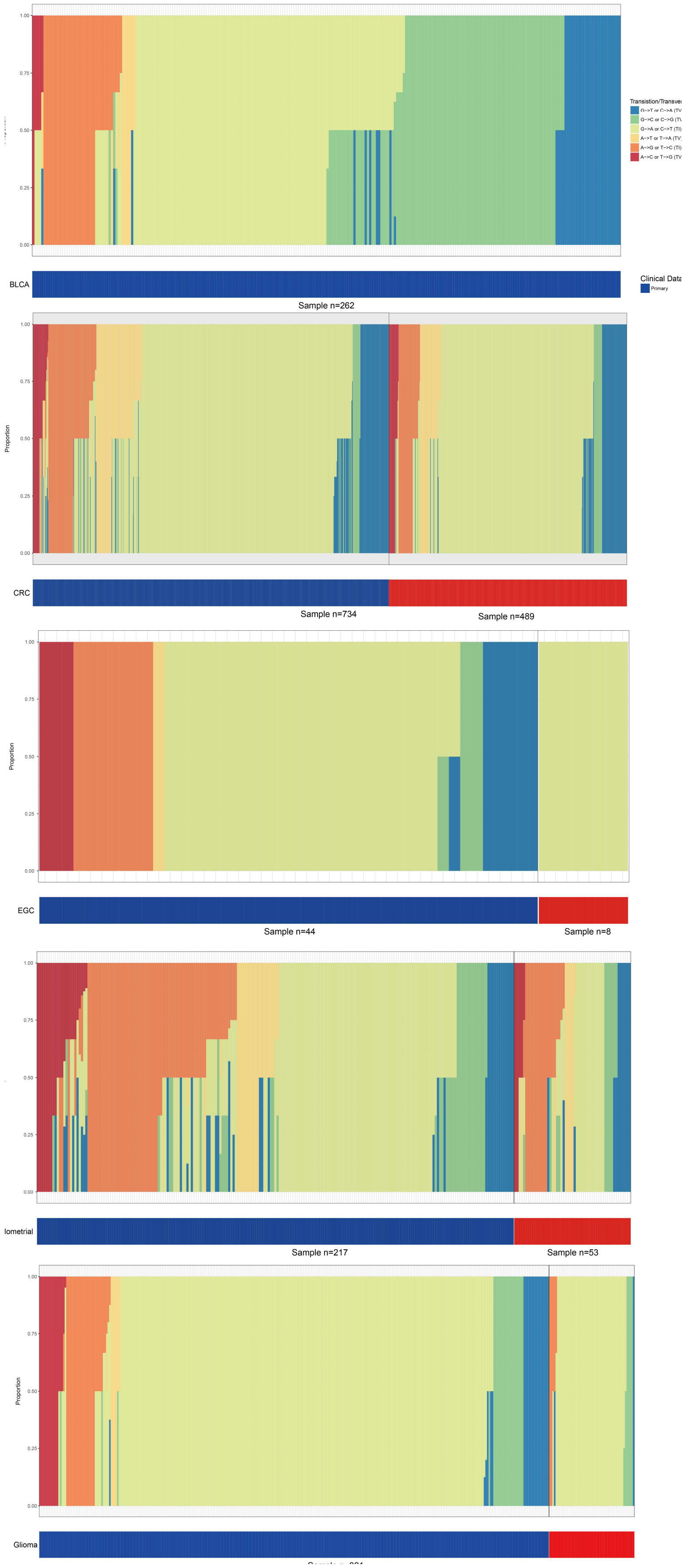

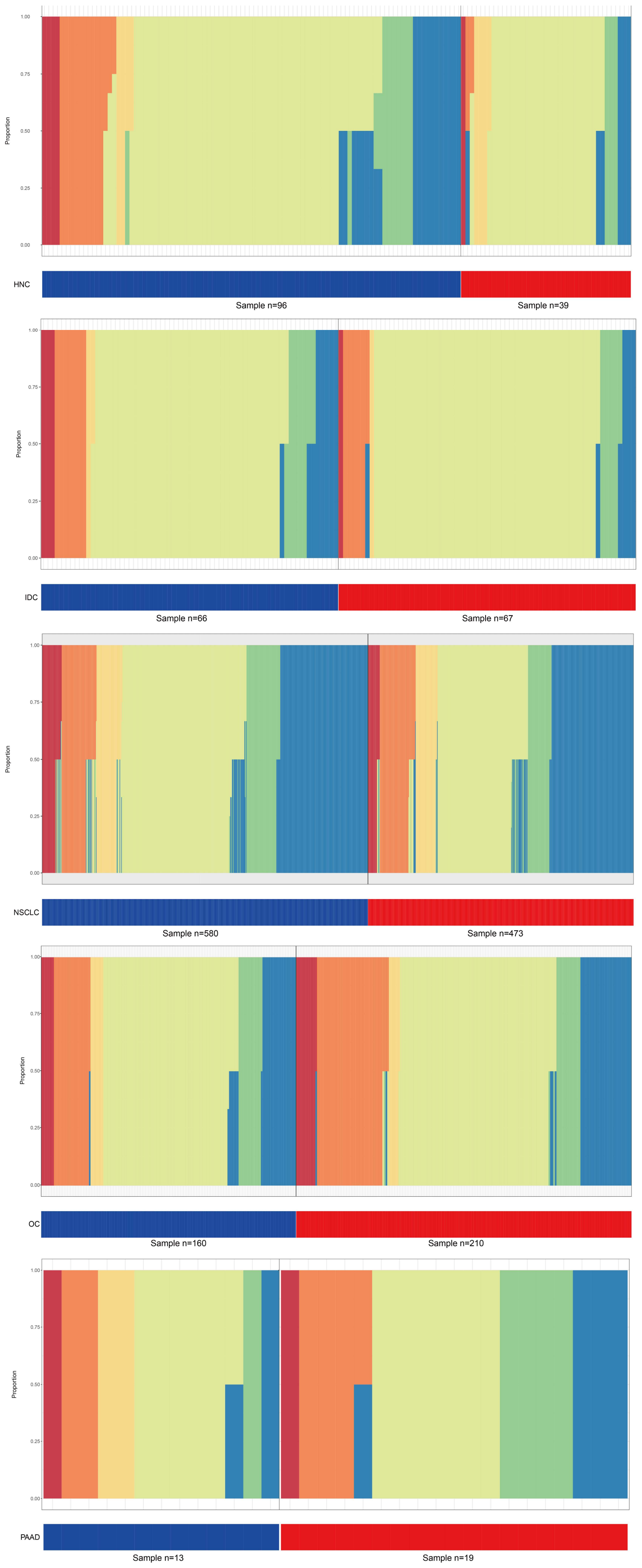

Proportion
1.00
0.75
0.50
0.25
0.00
HNC
Sample n=96
Sample n=39
IDC
Sample n=66
Sample n=67
NSCLC
Sample n=580
Sample n=473
OC
Sample n=160
Sample n=210
PAAD
Sample n=13
Sample n=19

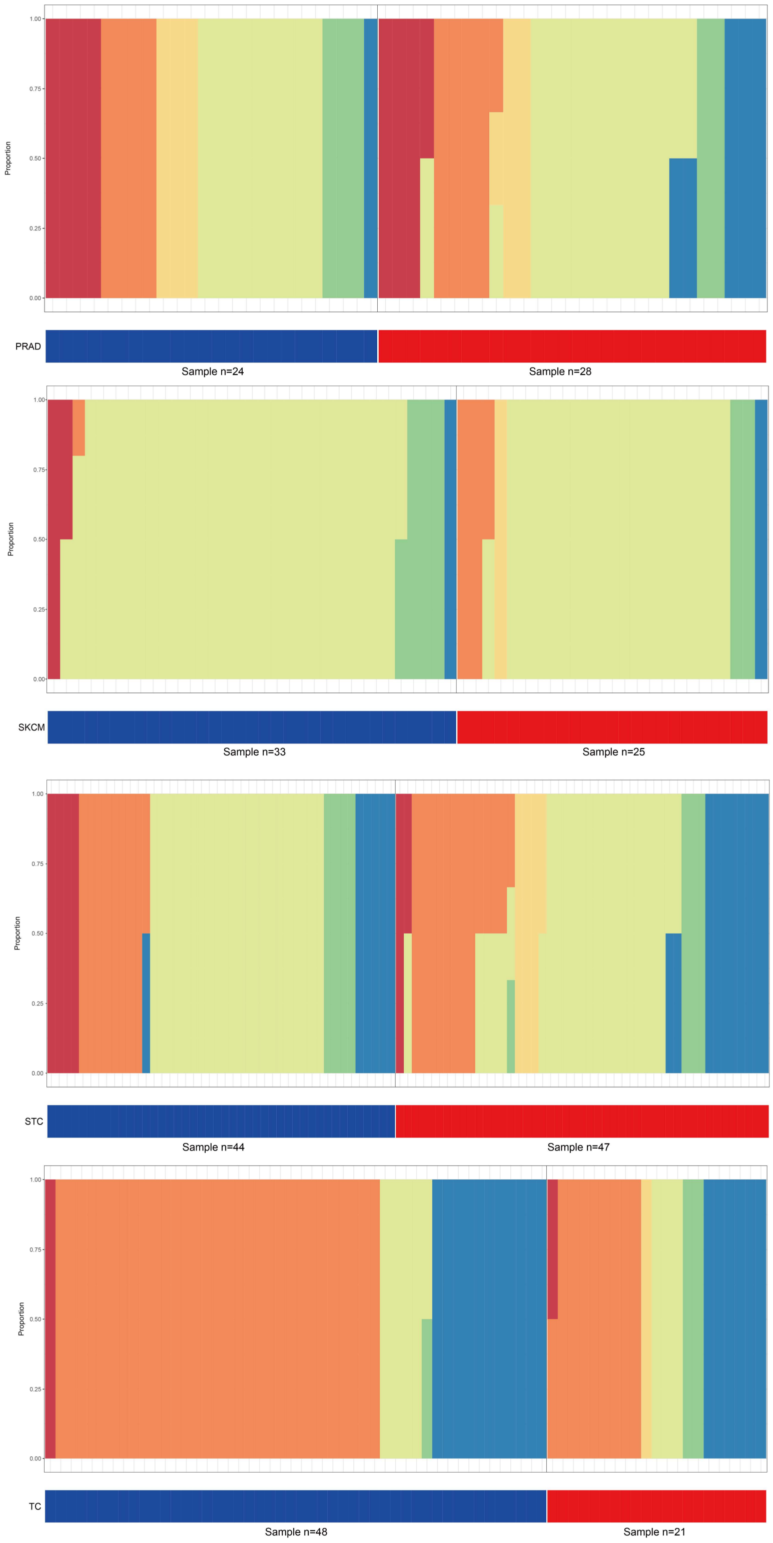

1.00
0.75
0.50
0.25
0.00
Proportion
PRAD
Sample n=24
Sample n=28
SKCM
Sample n=33
Sample n=25
STC
Sample n=44
Sample n=47
TC
Sample n=48
Sample n=21

Fig. S2. Circus plot of gene fusion in cancers.

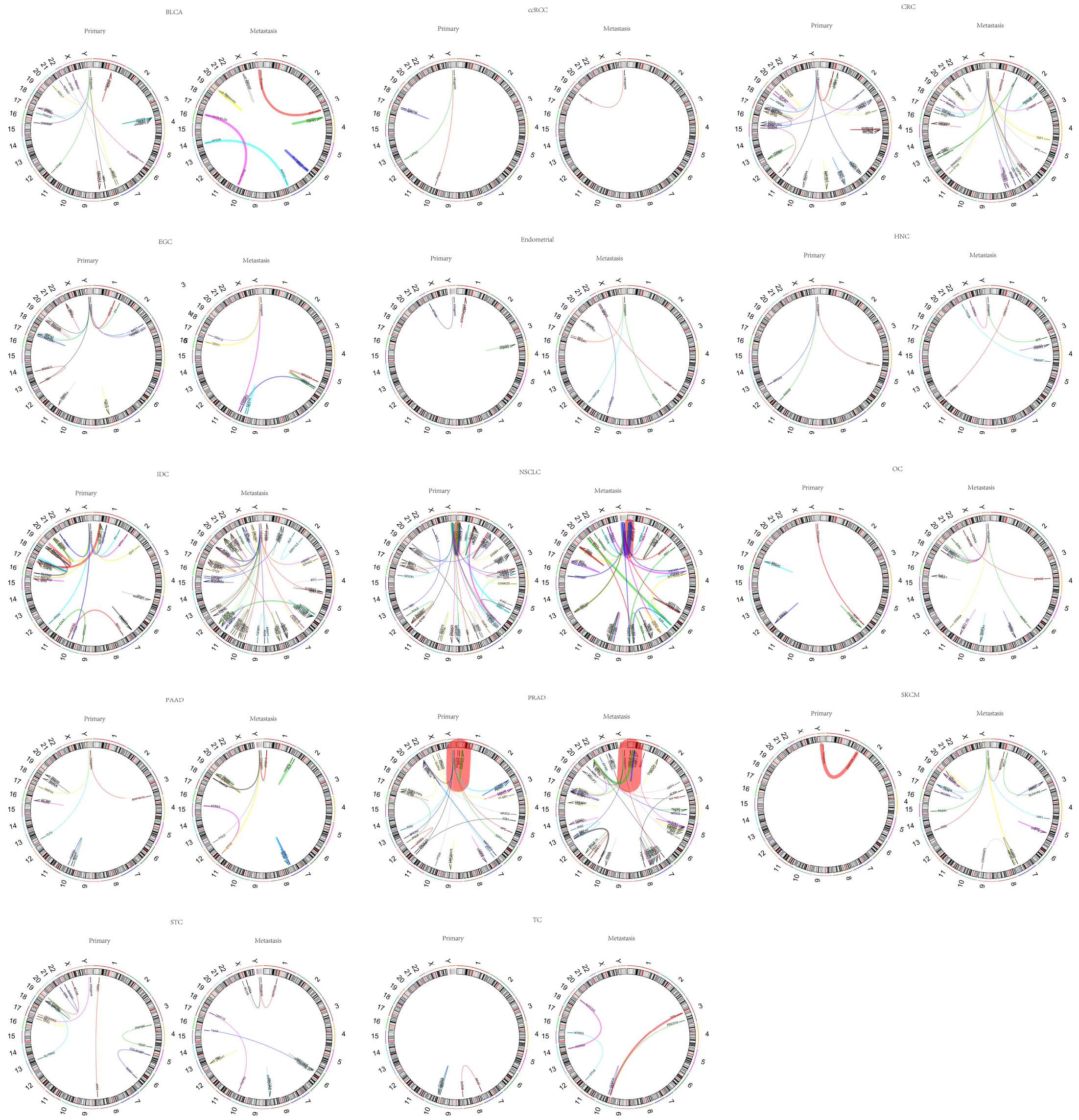

Fig. S3. Gene fusion difference between primary and metastatic tissues in cancers.

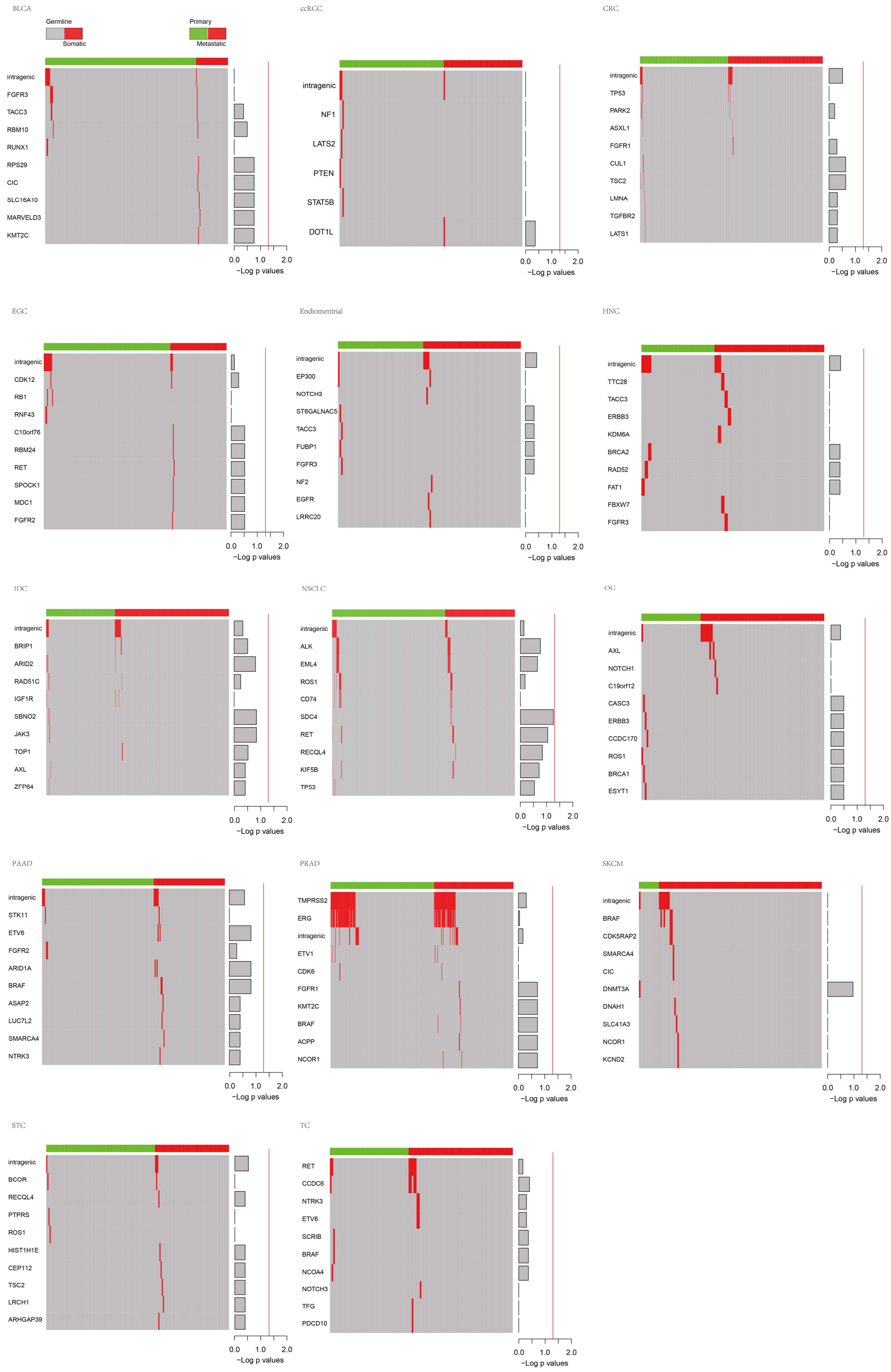

Supplement: Supplementary file 1 — Supplementary figures [file 41598_2017_13650_MOESM1_ESM.pdf]
